# Job stress and job satisfaction of physicians, radiographers, nurses and physicists working in radiotherapy: a multicenter analysis by the DEGRO Quality of Life Work Group

**DOI:** 10.1186/1748-717X-4-6

**Published:** 2009-02-06

**Authors:** Susanne Sehlen, Dirk Vordermark, Christof Schäfer, Peter Herschbach, Anja Bayerl, Steffi Pigorsch, Jutta Rittweger, Claudia Dormin, Tobias Bölling, Hans Joachim Wypior, Franz Zehentmayr, Wolfgang Schulze, Hans Geinitz

**Affiliations:** 1 Department of Radiotherapy and Radiooncology, University Munich, Munich, Germany; 2 Department of Radiotherapy and Radiooncology, University Würzburg/Halle, Würzburg, Germany; 3 Department of Radiotherapy and Radiooncology, University Regensburg, Regensburg, Germany; 4 Institute of Psychosomatic Medicine, Psychotherapy and Medical Psychology, Technical University Munich, Munich, Germany; 5 Department of Radiotherapy and Radiooncology, Vienna, Austria; 6 Department of Radiotherapy and Radiooncology, Technical University Munich, Munich, Germany; 7 Department of Radiotherapy and Radiooncology, University Halle, Halle, Germany; 8 Department of Radiotherapy and Radiooncology, University Frankfurt, Frankfurt, Germany; 9 Department of Radiotherapy and Radiooncology, University Münster, Münster, Germany; 10 Department of Radiotherapy and Radiooncology, General Hospital Landshut, Germany; 11 Department of Radiotherapy and Radiooncology, University Salzburg, Austria; 12 Department of Radiotherapy and Radiooncology, General Hospital Bayreuth, Bayreuth, Germany

## Abstract

**Background:**

Ongoing changes in cancer care cause an increase in the complexity of cases which is characterized by modern treatment techniques and a higher demand for patient information about the underlying disease and therapeutic options. At the same time, the restructuring of health services and reduced funding have led to the downsizing of hospital care services. These trends strongly influence the workplace environment and are a potential source of stress and burnout among professionals working in radiotherapy.

**Methods and patients:**

A postal survey was sent to members of the workgroup "Quality of Life" which is part of DEGRO (German Society for Radiooncology). Thus far, 11 departments have answered the survey. 406 (76.1%) out of 534 cancer care workers (23% physicians, 35% radiographers, 31% nurses, 11% physicists) from 8 university hospitals and 3 general hospitals completed the FBAS form (Stress Questionnaire of Physicians and Nurses; 42 items, 7 scales), and a self-designed questionnaire regarding work situation and one question on global job satisfaction. Furthermore, the participants could make voluntary suggestions about how to improve their situation.

**Results:**

Nurses and physicians showed the highest level of job stress (total score 2.2 and 2.1). The greatest source of job stress (physicians, nurses and radiographers) stemmed from structural conditions (e.g. underpayment, ringing of the telephone) a "stress by compassion" (e.g. "long suffering of patients", "patients will be kept alive using all available resources against the conviction of staff"). In multivariate analyses professional group (p < 0.001), working night shifts (p = 0.001), age group (p = 0.012) and free time compensation (p = 0.024) gained significance for total FBAS score. Global job satisfaction was 4.1 on a 9-point scale (from 1 – very satisfied to 9 – not satisfied). Comparing the total stress scores of the hospitals and job groups we found significant differences in nurses (p = 0.005) and physicists (p = 0.042) and a borderline significance in physicians (p = 0.052).

In multivariate analyses "professional group" (p = 0.006) and "vocational experience" (p = 0.036) were associated with job satisfaction (cancer care workers with < 2 years of vocational experience having a higher global job satisfaction). The total FBAS score correlated with job satisfaction (Spearman-Rho = 0.40; p < 0.001).

**Conclusion:**

Current workplace environments have a negative impact on stress levels and the satisfaction of radiotherapy staff. Identification and removal of the above-mentioned critical points requires various changes which should lead to the reduction of stress.

## Background

The Health care systems are undergoing major structural and financial changes. Ongoing changes to cancer care include an increase in the complexity of cases, available treatment options and better informed patients. One important new stressor is the increasing complexity of multimodal cancer treatment with difficulties for the individual health professionals to govern the treatment in all its details [1-6]. Especially in radiation oncology treatment has become progressively more complex within the past 10 to 15 years. Additional challenges are added with the growing proportion of cancer in the elderly caused by an augmented life expectancy in developed countries. At the same time health services restructuring and reduced public spending has lead to downsizing of hospital care services [7]. These factors contribute to an increased individual workload for the hospital staff.

Breaking bad news is one of a radiotherapists most difficult duties, yet medical education typically offers little formal preparation for this important task [8,9]. Without proper training, the discomfort and uncertainty associated with breaking bad news may lead physicians to emotionally distress.

Distress can lead to erosion of patient compliance which generates new distress for hospital staff [7]. [10]. In oncology additional strain is produced by the frequency of the deliverance of bad news and dealing with patient's death and suffering [11].

These imbalances with increasing demand of human and material resources on the one hand side and a lack of sufficient financial sources on the other side have produced a negative influence on the workplace environment and are potential sources of stress and burnout of cancer care workers in radiotherapy [12]. The impetus for the study was to analyze factors for stress and job satisfaction of cancer care workers within the context of different radiotherapy departments in Germany and Austria.

## Methods and study populations

### Recruiting of radiotherapy facilities

Radiotherapy facilities were recruited via the working group "quality of life" (Arbeitskreis "Lebensqualität") within the German Society of Radiation Oncology (DEGRO). Members of the working group were asked whether they were willing to locally carry out the study within their department ("local study coordinator"). Each local study coordinator was responsible for the information and mobilisation of the cancer care workers (physicians, radiographers, nurses, physicists) within his radiation oncology facility as well as for the distribution and recollection of the questionnaires. The local study coordinators were mailed a study protocol that provided guidelines for recruiting the participants and the questionnaires (see below). In addition they were asked to collect data on the clinic equipment, number of cancer care workers and patient load. The questionnaires could be allocated to the participating centre but not to the individual. For each hospital the works committees gave consent to proceed with the study. The study was carried out from August 2006 to February 2007.

### Questionnaires

Each cancer care worker was asked to give basic data on the category of her/his professional group, her/his age (four categories), gender, years of vocational experience (four categories), wether she/he was working night shifts or working on weekends and if she/he was getting free time compensation.

**Figure 1:**
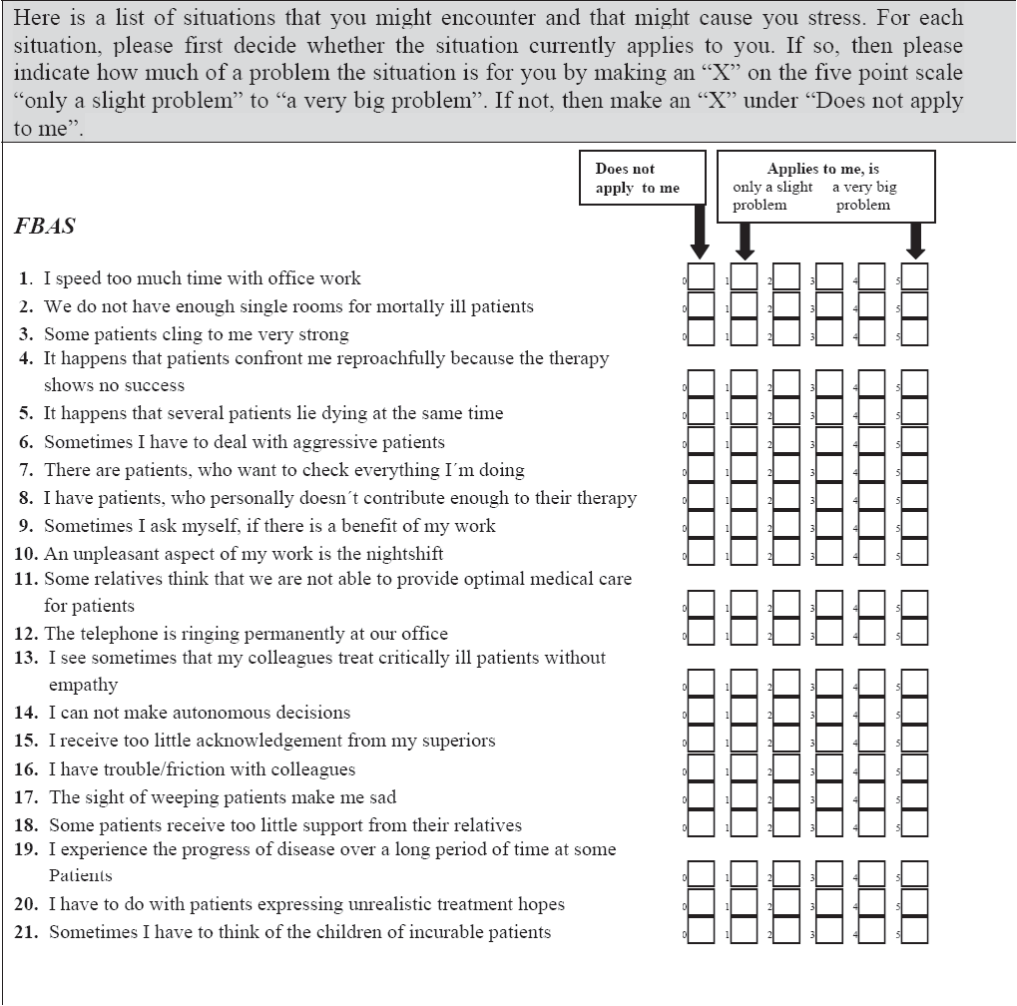
**" Stress Questionnaire of physicians and nurses (FBAS), Herschbach 1989**.

Job stress was evaluated with the "Fragebogen zur Belastung von Ärzten/Ärztinnen und Krankenpflegekräften" ("Questionnaire for Ascertaining Stress on Doctors and Nurses", Herschbach 1989 [13]). The validated questionnaire comprises 42 items. Each item was self-scored with the five categorized answers "not at all", "a little", "a little more", "quite a bit", or "a lot". Higher scores are associated with higher stress. The questionnaire is subdivided into 5 scales: "structural conditions" (e. g. "underpayment", "permanent ringing of the telephone"), "stress by compassion" (e.g. "against the conviction patients were kept alive with all resorts"), "problems with colleagues", "inconvenient patients" and finally "professional/private life" (e.g. "disruption of home life through spending long hours at work"). In addition a total score was built comprising of all 42 items (Fig. 1).

Global job satisfaction was evaluated with an ad hoc constructed one dimensional scale with nine categorical answers (1: very high job satisfaction to 9: total job dissatisfaction).

### Data analysis

The data analysis was carried out with the programme SPSS™ 14. for Windows. Influencing factors on job stress and satisfaction were analyzed using the Mann-Whitney Test or the Kruskall Wallis Test. Stepwise multiple linear regression analysis was performed for multivariate analyses. All tests were carried out two-sided. A p-level of 0.05 or below was considered to be significant.

## Results

11 radiotherapeutic treatment facilities in Germany and Austria participated in the study (8 universities, 3 general hospitals) comprising 534 cancer care workers. The overall response rate was 76.1% (n = 406), characteristics of the participants are given in table 1.

**Table 1:** Participants' characteristics

	**N (total n = 406)**	**percent**
**professional groups**		

physicians	82	22,7

nurses	113	31,2

radiographers	128	35,4

physicists	39	10,8

not available	44	

		

**gender**		

female	285	73,6

male	102	26,4

not available	19	

		

**age categories**		

20–29 years	93	23,4

30–39 years	113	28,5

40–49 years	120	30,2

50–59 years	65	16,4

≥ 60 years	6	1,5

not available	9	

		

**vocational experience**		

< 2 years	52	13,0

2-<5 years	65	16,3

5-<10 years	87	21,8

≥ 10 years	196	49,0

not available	6	

		

**work load**		

≤ 160 hours/months	225	58,3

> 160 hours/months	161	41,7

not available	20	

		

**Working night shifts**		

no	267	69,9

yes	115	30,1

not available	24	

		

**Free time compensation**		

no	116	28.9

yes	286	71.1

not available	4	

		

**Working on weekends**		

no	195	50,6

yes	190	49,4

not available	21	

		

**Night call/weekend call duties**		

no	304	80,4

yes	74	19,6

not available	28	

### Job stress

Nurses and physicians showed the highest levels of job stress (mean FBAS total score 2.2 and 2.1, respectively), whereas radiographers (mean total score 1.7) and physicists (mean total score 1.0) disclosed lower levels of job stress (p < 0.001) (table 2). For physicians, nurses and radiographers the highest stress rates were caused by "structural conditions" and "stress by compassion" (table 2). Physicists reported in all low stress levels with the highest score values in the scales "structural conditions" and "problems with colleagues". On the item level the four greatest sources of physician's job stress were" too much office work" (mean score 3.4), "time pressure" (mean score 3.36), "ill-defined responsibilities" (mean score 3.13) and "breaking off the conversation with the patient" (mean score 3.10). For nurses the greatest stress factors stemmed from "permanent ringing of telephone" (mean score 3.53), "against the conviction patients were kept alive by all means" (mean score 3.22), "underpayment" (mean score 3.21) and "time pressure" (mean score 3.11). Radiographers rated the following items as the most stressing: "against the conviction patients were kept alive by all means" (mean score 2.88), "stress due to patient's disease progression" (mean score 2.79), "high physical workload" (mean score 2.76) and "patients suffering of my therapy" (mean score 2.74). Physicists expressed as sources of stress "time pressure" (mean score 2.82), "underpayment" (mean score 2.34), "ill-defined responsibilities" (mean score 2.19) and "reduction of private life through high workload" (mean score 2.16) (table 3).

**Table 2:** FBAS – stress items and profession

**Item**	**Total**	**Physician** Mean SD	**Nurse** Mean SD	**Radiographer** Mean SD	**Physicist** Mean SD
**"too much office work"**	2.15	**3.40**	2.39	1.21	2.15
	
	± 1.73	± **1.42**	± 1.69	± 1.48	± 1.45

**"having conflicting demands on the time"**	2.95	**3.36**	**3.11**	2.70	**2.82**
	
	± 1.62	± **1.47**	± **1.76**	± 1.57	± **1.50**

**"illdefined responsibilities"**	2.57	**3.13**	2.78	2.15	**2.19**
	
	± 1.67	± **1.52**	± 1.60	± 1.73	± **1.65**

**"breaking off a conversation with the patient"**	2.40	**3.10**	2.80	2.17	0.31
	
	± 1.68	± **1.54**	± 1.52	± 1.60	± 0.87

**"disruption of home life through spending long hours at work"**	1.88	2.82	1.31	0.88	**2.16**
	
	± 1.89	± 1.93	± 1.66	± 1.54	± **1.78**

**"underpayment"**	2.89	3.07	**3.21**	2.64	**2.34**
	
	± 1.74	± 1.74	± **1.67**	± 1.74	± **1.74**

**"permanent ringing of telephone"**	2.70	2.76	**3.53**	2.26	1.76
	
	± 1.74	± 1.78	± **1.46**	± 1.83	± 1.62

**"against the conviction patients were kept alive with all resorts"**	2.45	1.37	**3.22**	**2.88**	0.70
	
	± 1.88	± 1.62	± **1.79**	± **1.69**	± 1.16

**"stress due to patient's disease progression"**	2.71	2.71	2.93	**2.79**	1.21
	
	± 1.42	± 1.35	± 1.41	± **1.21**	± 1.62

**"high physical workload"**	2.18	1.16	2.84	**2.76**	0.64
	
	± 1.66	± 1.38	± 1.69	± **1.33**	± 0.90

**"patients suffering of my therapy"**	2.20	1.93	2.23	**2.74**	0.42
	
	± 1.67	± 1.51	± 1.77	± **1.53**	± 1.09

**Table 3:** FBAS scales/total score and job stress

**scale**		**Mean**	**Standard deviation**	**Significance**
**structural conditions**	**physician**	2.5856	.98258	
	**nurse**	2.7603	1.13287	**P < 0.001**
	**radiographer**	2.0297	.94769	
	**physicist**	1.4447	.91103	

**compassion**	**physician**	2.1598	.85505	
	**nurse**	2.2913	.98817	**P < 0.001**
	**radiographer**	2.0265	.79141	
	**physicist**	.8518	.75022	

**inconvenient patients**	**physician**	2.0434	.88469	
	**nurse**	2.1789	1.02272	**P < 0.001**
	**radiographer**	1.5164	.78164	
	**physicist**	.3110	.65245	

**job/private life**	**physician**	1.8317	1.35409	
	**nurse**	1.5705	1.31611	**P < 0.001**
	**radiographer**	.5515	.80870	
	**physicist**	1.0128	.97877	

**problems with colleagues**	**physician**	1.7175	1.02362	
	**nurse**	1.7637	1.07077	**n.s**.
	**radiographer**	1.8832	1.08493	
	**physicist**	1.4808	1.15781	

**total score**	**physician**	2.1368	.78242	
	**nurse**	2.2125	.89627	**P < 0.001**
	**radiographer**	1.7320	.70041	
	**physicist**	.9616	.64292	

Besides professional group the following variables were tested for their association with the FBAS total stress score and with 5 FBAS scales: age category (20-<30, 30-<40, 40-<50, 50-<60, ≥ 60 years) gender, vocational experience (<2, 2-<5, 5-<10, ≥ 10 years), work load (≤ 160 vs. > 160 hours/months), working night shifts (yes vs. no), Night call/weekend call duties (yes vs. no), working on weekends (yes vs. no) and possibility of free time compensation (yes vs. no). In univariate analysis the the following variables were associated with more job stress: **total FBAS score: **working night shifts (p < 0.001) and working on weekends (p < 0.001); **"structural conditions": **working night shifts (p < 0.001), working on weekends (p < 0.001) and no free time compensation (p = 0.013); **"stress by compassion": **female gender (p = 0.038), working night shifts (p < 0.001) and working on weekends (p < 0.001); **"problems with colleagues": **age < 50 years (p = 0.024); **"inconvenient patients": **working night shifts (p < 0.001) and working on weekends (p < 0.001); **"professional/private life": **male gender (p = 0.006), working night shifts (p < 0.001), Night call/weekend call duties (p < 0.001), working on weekends (p < 0.001), no free time compensation (p < 0.001) and working more than 160 hours/months (p = 0.001).

**Figure 2:**
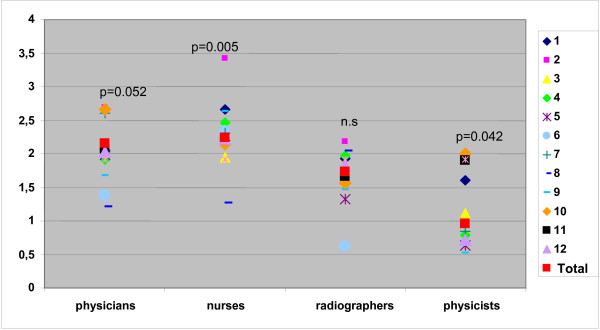
**Total score of job stress- profession group and clinic n.s not significant**.

Comparing the total stress scores of the hospitals and job groups we found significant differences in nurses (p = 0.005) and physicists (p = 0.042) and a borderline significance in physicians (p = 0.052) (Figure 2).

In addition to the above mentioned variables the hospital was included in the multivariate analyses. The following parameters gained significance: **total FBAS score: **professional group (p < 0.001), working night shifts (p = 0.001), age group (p = 0.012) and free time compensation (p = 0.024); **"structural conditions": **professional group (p < 0.001), working on weekends (p = 0.005) and working night shifts (p = 0.042); **"stress by compassion": **professional group (p < 0.001), no free time compensation (p < 0.001) and age group (p = 0.032); **"problems with colleagues": **age group (p = 0.046); **"inconvenient patients": **professional group (p < 0.001), age group (p < 0.001), no free time compensation (p < 0.001) and working night shifts (p < 0.001); **"professional/private life": **working on weekends (p = 0.002), working night shifts (p = 0.003), professional group (p = 0.015) and no free time compensation (p = 0.005).

### Job satisfaction

**Figure 3:**
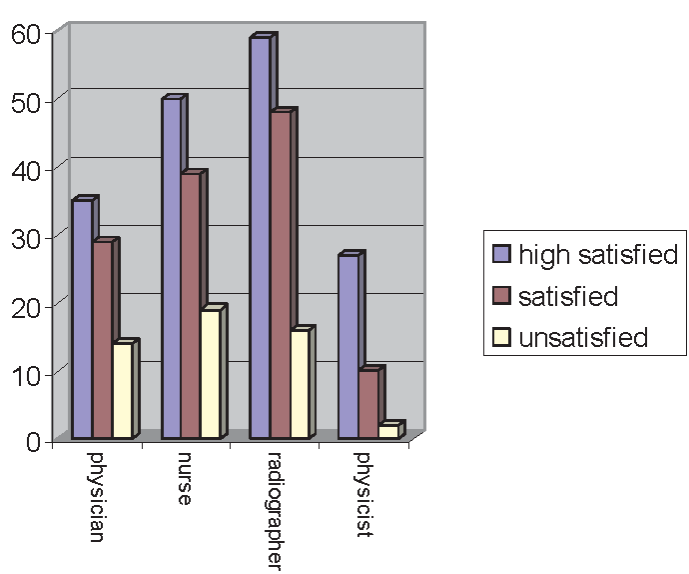
**Satisfaction and professional group**.

Like job stress satisfaction was associated with professional group: physicists had the highest satisfaction values whereas the other professional groups had clearly lower levels without much difference in between the three groups (figure 3). There were no other factors that were associated with job satisfaction in univariate analyses.

In multivariate analyses "professional group" (p = 0.006) and "vocational experience" (p = 0.036) were associated with job satisfaction, with cancer care workers with less than two years of vocational experience having a higher global job satisfaction. The total FBAS score correlated with job satisfaction (Spearman-Rho = 0.40; p < 0.001).

## Discussion

In this paper we report on job stress and job satisfaction of cancer care workers in radiation oncology clinics in Germany and Austria. Although the sample of hospitals is not representative for all radiation therapy facilities in both countries the collected data fits to previously published reports in other countries. This is the first published survey of its kind conducted in German speaking countries. Considering the high response rate the data should adequately mirror job stress in the 11 participating hospitals and could serve as a source for generating hypotheses. Since nearly three quarters of the participating centres were university hospitals extrapolation to non-university facilities should be carried out with caution.

The findings of our study indicate that job stress levels vary between professional groups. Physicians and nurses rated their job stress higher than radiographers and medical physicists. Job stress also stemmed from different sources in between professional groups: physicians, nurses and radiographers were mostly stressed by structural conditions and compassion while physicists were stressed -although by a much lower level- by structural conditions and problems with colleagues. This is in line with the lower patient contacts of physicists in routine clinical work. Since the total stress score correlated with satisfaction medical physicists also disclosed higher job satisfaction levels than the other professional groups.

With the aging of the population there will be a growing demand to recruit health care professionals -especially in oncology. On the other hand birth rates are low almost all over the European Union [14]. and will most likely result a shortage in skilled personnel within the next years. The health care system has to find ways to attract young people to find their professional career within this system and -almost as important- to provide conditions that they stay within this vocation. Job stress is an important factor for cancer care workers to consider alternative work situations [15].

Job stress in itself is not only disturbing for the working health care individual but can also have a profound effect on the interaction with the patient considering that patient in oncology, especially in radiotherapy, have a high stress level distress [16-18]. Increasing evidence suggests that physician distress can lead to erosion of physician compassion [1,19], patient compliance [10] and the quality of care physicians deliver [1,20]. Physicians under stress are reported to have a higher tendency in treating patients poorly both medically and psychologically [21]. They are also more likely to make errors of judgement.

Personal, interpersonal and organisational factors have been reported to relate to job stress. One of the organisational factors that required a highly increased workload from health professionals in the past years is documentation. Einhorn et al. conducted a postal survey in 2.493 US oncologists [22]. They report that increased documentation caused the greatest concern among respondents and negatively influenced job satisfaction. More than 40% reported that high documentation workload lead to diminished patient care and decreases in teaching (48%) and research (39%). In concordance with the results of Einhorn et al. [22]. physicians in our study ranked "too much office work" as the highest job stressor greatly surpassing other factors commonly thought to be associated with job strain in oncology like "stress due to patient's disease progression".

Further important structural conditions that caused high stress among participants were time pressure ("having conflicting demands on the time", "breaking off a conversation with the patient" and "permanent ringing of the telephone") as well as "underpayment" and "high physical workload". Grunfeld et al. carried out a survey in 681 cancer care workers in Ontario [15]. They found that "having too great volume of work", "having inadequate staffing to do the job properly", ""feeling under pressure to make deadlines" and "having conflicting demands on time" were mayor derminants of job stress. Ernst and colleagues surveyed 249 pediatric nurses and found that pay was one of the mayor determinants of job stress [23].

Cancer care workers in our study reported more job distress when they were working night shifts, and weekends or were not getting free time compensation for working long hours. Data from Ǻrkerstedt et al. support the notion that night time work is hazardous to a persons' long term well being [24]. For physicians, nurses and radiographers "structural conditions" and "patient-compassion" were the major causes of their stress. Documentation/paperwork decreases the ability of cancer care workers to spend time with their patients. Growing incidence of stress by medical specialists can be caused by recent changes in society. Patients are better informed, more critical and better protected by law [25]. In addition job security has diminished and plays a major role. Grunfeld et al. [15]. in their analysis of 681 cancer care workers in Canada disclosed that job stress increased with workload. To reduce job stress of cancer care workers in radiation oncology measures should be undertaken to improve the structural conditions within the departments. Such measures could be: better definitions of responsibilities for the individual cancer care worker, delegation of office work to other professional groups (e. g. data managers, secretaries), optimization of work processes (quality management) and training of communication skills and conflict solving strategies of all professional groups. Several authors showed for example that stress for hospital nurses correlated with conflict of doctors [26]. They have to accept that death is an intrinsic factor of their profession. Thus cancer care workers have to learn to function at an optimal emotional and intellectual level despite such strong stressors [27]. A better balance may be obtained between time spent at work and time spent at home.

Stress by compassion and inconvenient patients were higher among nurses than among physicians and radiographers. In agreement with other investigators we found factors that may be greater sources of stress for women physicians [28]. "The cancer care workers in this survey felt that the mean level of stress in dependence of years of vocational experience was similar. These response suggest that the stress does not get better after completion of training. Efforts to debunk the myth of "things getting better" early in training and instead emphasize the importance of developing balance and strategies for promoting personal wellness may be warranted" [16].

Although the response rate is high for a physician survey, response bias remains a possible confounding factor [29]. Objective job stress like the actual number of hours the participants had to work or if they were on temporary employment was not directly measured in this survey but the fact that subjective job stress correlated with working night shifts and working weekends does indicate that both measures -objective and subjective- are closely related.

Job stress in this sample of cancer care workers in radiation oncology departments is highly determined by structural conditions followed by problems related to patient compassion. As in Germany and Austria health care workers and in particular physicians are in short supply opinion leaders in health care politics and hospital administrators should try to focus their attention on how to improve structural conditions and job satisfaction for this group of professionals. Besides of accepting job stress as a problem in the field of health care future studies and strategies might encompass a reduction of the individual work load, optimization of work processes, a shift of office work onto other professional groups, training of communication and conflict solving skills and strategies for promoting personal wellness and an even balance of professional and private life.

## Competing interests

The authors declare that they have no competing interests.

## Authors' contributions

SS and HG conceived of the study, and participated in its design and coordination, performed the statistical analysis and drafted the manuscript. DV and CS conceived of the study, participated in its design and coordination, carried out the analysis in the centres and drafted the manuscript. AB, SP, JR, CD, TB, HJW, FZ and WS carried out the analysis in the different centres. PH conceived of the study, participated in its design and drafted the manuscript.
